# Assessing spatiotemporal characteristics of atmospheric water cycle processes over the Tibetan Plateau using the WRF model and finer box model

**DOI:** 10.1038/s41598-024-55208-0

**Published:** 2024-02-29

**Authors:** Xiaoduo Pan, Xiaowei Nie, Hu Li, Rana Muhammad Ali Washakh, Jing Jin

**Affiliations:** 1 National Tibetan Plateau Data Center (TPDC), State Key Laboratory of Tibetan Plateau Earth System, Environment and Resources (TPESER), Institute of Tibetan Plateau Research, Chinese Academy of Sciences, Beijing, 100101 China; 2 Science Center of Lingshan Forum of Guangdong Province, Guangzhou, 511466 China; 3 University of the Chinese Academy of Sciences, Beijing, 100049 China; 4 School of Environment and Natural Resources, Renmin University of China, Beijing, 100872 China; 5 School of Ecology and Environment, Tibet University, Lhasa, 850000 China

**Keywords:** Water vapor transport, Weather research and forecasting model, Moisture recycling, Tibetan Plateau, Atmospheric science, Climate change

## Abstract

The Tibetan Plateau (TP) is the highest and one of the most extensive plateaus in the world and serves as a hotspot of climate change. In the context of climate warming, changes in evapotranspiration (ET) and external water vapor transport have a significant impact on assessing atmospheric water cycle processes over the TP. By using the Weather Research and Forecasting (WRF) model for long-term simulations and the finer box model for the calculation of water vapor along the boundary of the TP, the external atmospheric water vapor transport and its spatiotemporal characteristics over the TP are finely described. The simulated precipitation and ET are well-simulated compared with observation. Research results show that: (1) The total water path on the TP decreases from southeast to northwest. Water vapor is mainly transported into the TP from the western and southern boundaries. The net water vapor flux transported from the western boundary to the TP by westerly wind is negative, while the net water vapor flux transported from the southern boundary to the TP by southerly wind is positive. (2) In spring and winter, water vapor is mainly transported into the TP by mid-latitude westerlies from the western boundary. In summer, water vapor transport controlled by mid-latitude westerlies weakens, and water vapor is mainly transported into the TP from the southern boundary. In autumn, water vapor controlled by mid-latitude westerlies gradually strengthens, and water vapor is mainly transported into the TP from the western boundary. In addition, the ratio of ET to precipitation on the TP is about 0.48, and the moisture recycling is about 0.37. Water vapor mainly comes from external water vapor transport.

## Introduction

The Tibetan Plateau (TP), which has an area of about 2.5 million km^2^ and an average elevation of over 4000 m. It is the highest and one of the most extensive plateaus in the world. Together with the South Pole and the North Pole, it is known as the “three poles” of the earth^1–3^. Due to its specific underlying surfaces and unique terrain, the TP is comprehensively influenced by multiple climatic systems, including the mid-latitude westerlies, the Indian summer monsoon, and the East Asian monsoon, and is the “amplifier” of global climate change^1,3–5^. The TP is renowned for its abundant water resources, known as the “Asian water tower (AWT)”, with its lake area and glaciers accounting for 52% and 80% of the total in China, respectively^6,7^. The TP is also the source of major Asian rivers including the Yangtze River, Yellow River, and Lancang River, providing water resources for industry, agriculture, and production to approximately 40% of the world’s population^6,8,9^. The water vapor budget of the TP has a direct impact on the precipitation, thereby changing the surface evaporation and runoff, and has important impacts on the water cycle process of the plateau and its surrounding regions^10–12^. Therefore, it is crucial to comprehend the spatiotemporal characteristics of the atmospheric water cycle processes on the TP.

Extensive research has been conducted on the atmospheric water cycle processes on the TP^13–17^ and many key datasets have been produced and stored in the National Tibetan Plateau Data Center (TPDC)^18^, such as the dataset of oceanic moisture contributions to the precipitation over the TP simulated by the Water Accounting Model-2layers (WAM-2layers) during 1979–2015^19^. Generally, atmospheric water vapor originates from moisture recycling within the local region and advection from remote regions^20,21^. The water vapor that leads to precipitation on the TP mainly comes from the remote water vapor transport controlled by the Asian monsoon system and the mid-latitude westerlies, as well as moisture recycling on the TP^22^. Feng and Zhou^23^ proposed that the majority of water vapor on the TP is transported from the southern boundary of the TP, followed by the western boundary, and the amount of water vapor transported from the western boundary is approximately 32% of that transported from the southern boundary. Based on the modified WAM, Zhang et al.^24^ estimated that over 69% of the water vapor contributing to precipitation on the TP originates from land, while over 21% originates from the ocean, and the water vapor transported by the mid-latitude westerlies and the Indian summer monsoon contributes the most to precipitation on the TP. Based on the WAM-2layers to quantify the moisture source–sink relations, Zhang et al.^25^ pointed out that the main sources of water vapor for precipitation in the southern and northern regions of the TP are different, and the precipitation in the northern region of the TP mainly comes from the northwest source (extending from the plateau to Europe), accounting for 38.9%, while the precipitation in the southern region of the TP mainly comes from the southeast source (extending from the southeastern of the TP to the Indian Ocean region), accounting for 51.4%.

In the context of global warming, the climate of the TP has undergone significant changes, and the uncertainty of the atmospheric water cycle processes has increased. Studies have shown that the temperature of the TP rises faster than other regions at the same latitude, with an increased rate that is twice the global mean temperature increase^26,27^. With the increase in temperature, the near-surface state of the TP has undergone significant changes, including glacier melting, lake expansion, permafrost degradation, and vegetation changes. These changes will lead to an increase in evapotranspiration (ET) on the TP, further affecting the land–atmosphere interaction of the TP, and thus influencing the atmospheric water cycle processes, resulting in an increase in total water path and precipitation^17,28–31^. In addition, some studies have indicated a weakening trend in the Indian summer monsoon over the past few decades^9,32,33^, resulting in a reduction of water vapor transported from the Indian Ocean to the TP and leading to a decrease in precipitation in the southern region of the TP^34^. It is important to note that the changing trends of water vapor are different in the sub regions of the TP. Significant changes have occurred in the water vapor content from different sources on the TP, the spatiotemporal characteristics and mechanism of the atmospheric water cycle process on the TP need to be further studied and assessed.

The impact of global climate change on atmospheric water cycle processes leads to the redistribution of atmospheric water in time and space^7^, which is crucial for precipitation, surface water resources, and the ecological environment. However, due to the limitation of data on the TP, it is difficult to accurately study the characteristics of water vapor transport on the TP and its surrounding regions. The Weather Research and Forecasting (WRF) model is a numerical weather prediction and atmospheric simulation system that has been widely used to simulate the atmospheric water cycle processes over the TP^35–38^. Gao et al.^39^ showed that the WRF model performs better than ERA-Interim reanalysis in reproducing the interannual and long-term trends of precipitation over the TP. Jiang et al.^40^ investigated the simulation of summer precipitation over the TP from 1979 to 2010 using the WRF model at different horizontal resolutions (50 km and 15 km) and found that increasing the horizontal resolution significantly improved the model’s ability to simulate climatological mean rainfall. Moreover, using the WRF model at a horizontal resolution of 9 km, Zhou et al.^41^ conducted a long-term simulation (2000–2019) over the TP and demonstrated that the WRF model can effectively capture the interannual and seasonal variations in precipitation and reduce the biases compared with the reanalysis data.

The open research question on water vapor transport over the TP is that the complex topography significantly affects the water vapor transport process, especially water vapor transport from the southern boundary, and high-resolution simulations are expected to provide a better analysis of the water vapor transport process over the TP. This study hypothesizes that higher-resolution representations of TP topography in simulations lead to more accurate depiction of barriers and channels, thereby impacting the patterns of water vapor transport. Specifically, we anticipate that finer resolution will result in a more realistic representation of very high peaks as barriers, potentially reducing the influx of water vapor through the southern boundaries. By utilizing the WRF model, we conducted long-term, high-resolution simulations to capture the dynamics of water vapor transport over the TP. The finer box model is employed to quantitatively assess the spatiotemporal characteristics of water vapor transport from four boundaries, thereby providing important insights into the impact of climate change on the atmospheric water cycle processes on the TP.

## Data and method

### WRF model configure

**Figure 1:**
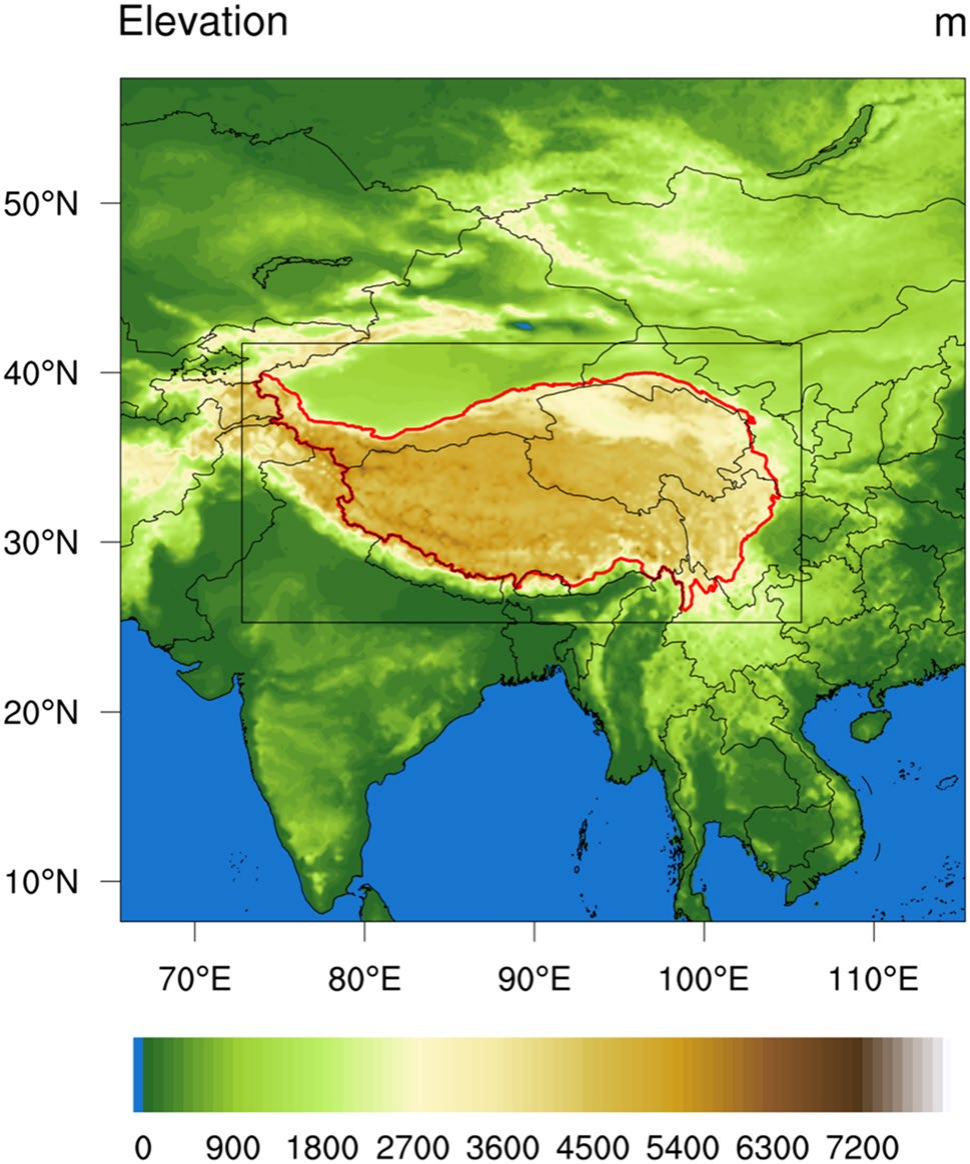
The study area. The outer black border represents the outer domain. The inner black border represents the inner domain. The red boundary represents the Tibetan Plateau.

The Advanced Research WRF Model Version 3.7.1 is used in this study, which is a non-hydrostatic model, including Arakawa C-grid staggering for a horizontal grid, a fully compressible system of equations, and a third-order Runge–Kutta scheme for time-split integration^42^. In this study, two nested domains are employed in this study, with the outer domain (Domain 1) consisting of 200 × 200 grid points at a horizontal resolution of 0.25° and the second nested domain (Domain 2) consisting of 300 × 600 grid points at a horizontal resolution of 0.05° (Fig. 1). The Purdue Lin Scheme^43^ is the microphysics option, the Kain–Fritsch scheme^44^ is the cumulus convection parameterization option, the Yonsei University scheme^45^ is the planetary boundary layer (PBL) option, the Unified Noah Land Surface Model (LSM)^46^ as the land-surface model option, and the Dudhia scheme^47^ as the radiation option are used in this simulation (Table 1). The outer domain is forced with the reanalysis datasets of the Final Analysis (FNL) from the National Center for Environmental Prediction (NCEP)^48^ and provides initial and boundary conditions for the second nested domain.

**Table 1:** Physical configuration of the WRF model used in this study.

Physics processes	Domain 1	Domain 2
Horizontal	201 × 201	301 × 601
Time interval	150 s	30 s
Projection	lat-lon	lat-lon
Horizontal resolution	0.25°	0.05°
Microphysics	Lin et al. scheme	Lin et al. scheme
Cumulus	Kain–Fritsch	Kain–Fritsch
PBL	YSU	YSU
Radiation	Dudhia	Dudhia
Surface-Land	Noah LSM	Noah LSM
Initial and Boundary data	NCEP-FNL Reanalysis	Domain 1

### Water vapor transport and finer box model

The box model is a commonly used approach for estimating water vapor flux by considering the entire research region as a single box. However, the accuracy of this method is limited as it oversimplifies the complex boundaries within the study area, resulting in essentially useless of high-resolution atmospheric simulation data. To account for the boundary complexity of the study area and improve the accuracy of the net inflow of water vapor through the boundary of the study area, the “finer box model” developed by Pan et al.^37^ is employed to involve a more detailed calculation of water vapor at each grid point along the boundaries of the study area. Instead of treating the entire region as a single box, the “finer box model” takes into account the individual contributions of water vapor from multiple grid points along the boundaries. The water vapor flux at each grid point is calculated along the red boundary in Fig. 1. The “finer box model” is a refinement of the traditional box model, and it aims to enhance the accuracy of estimating the net inflow of water vapor through the boundary by leveraging high-resolution atmospheric simulation data. The equation for calculating the water vapor flux for each grid is as follows:

1 $${\overrightarrow{q}}_{(i,j)}=\frac{1}{g}{\int }_{0}^{{p}_{s}} \left({\overrightarrow{W}}_{(p,i,j)}{q}_{(p,i,j)}\cdot {\overrightarrow{n}}_{(i,j)}\right)dp$$

where $i$ and $j$ represent the location identification of the boundary grid in the whole study area. $q$ is the specific humidity; $p$ is the pressure; and ${p}_{s}$ is the pressure at the top of the atmosphere. $g$ is the gravitational constant. $\mathop{W}\limits^{\rightharpoonup}$ is the wind component at $x,y,z$ directions. $\overrightarrow{n}$ is the unit normal vector at the boundary. The unit normal vector at each grid point along the boundary represents the perpendicular direction to the boundary surface. By using the unit normal vector at each grid point at the boundary, it can be determined whether water vapor is transported into or out of the TP, and more accurately calculate the changes in the water vapor transport process on the TP.

The equation for regional net water vapor flux ($\mathrm{\Delta q}$) is as follows:

$$\mathrm{\Delta q}=\sum {q}_{(i,j)}$$

2 $$\begin{array}{c}0\le i\le M\\ 0\le j\le N\end{array}$$

where $M$ and $N$ represent the sizes of the used grids in the adopted unit of space. In addition, the equation for regional moisture recycling rate is as follows:

3 $$\gamma =\frac{P-{C}_{q}}{P}$$

where ${C}_{q}$ is the net inflow of atmospheric water,$P$ is the precipitation.

### Validation data and verification indicators

The Multi-Source Weighted-Ensemble Precipitation (MSWEP)^49^ and Global Land Evaporation Amsterdam Model (GLEAM)^50^ are employed to evaluate the simulated precipitation and ET in this study. The MSWEP integrates gauge, satellite, and reanalysis data to evaluate the simulated precipitation, with a temporal resolution of 3 h and a spatial resolution of 0.25°, covering the period from 1979 to 2015. The GLEAM combines satellite observations to develop a global evapotranspiration product for near real-time applications, with a temporal resolution of daily and a spatial resolution of 0.25°, ranging from 1980 to 2015, which is used to evaluate the simulated ET.

The performance of the WRF simulation is assessed using the root mean square error (RMSE), mean absolute error (MAE), and correlation coefficient (CC). The equations for the evaluation metrics are as follows:

4 $${\text{RMSE}}=\sqrt{\frac{1}{n}\sum_{i=1}^{n} {\left({S}_{i}-{O}_{i}\right)}^{2}}$$

5 $${\text{MAE}}=\frac{1}{n}\sum_{i=1}^{n} \left|{S}_{i}-{O}_{i}\right|$$

6 $${\text{CC}}=\frac{cov\left(S,O\right)}{{\sigma }_{S}{\sigma }_{O}}$$

where, $S$ represents the WRF simulated value. $O$ represents the observed value. $cov$ is the covariance. ${\sigma }_{S}$ is the standard deviation of $S$. ${\sigma }_{O}$ is the standard deviation of $O$.

## Results

### Model validation

**Figure 2:**
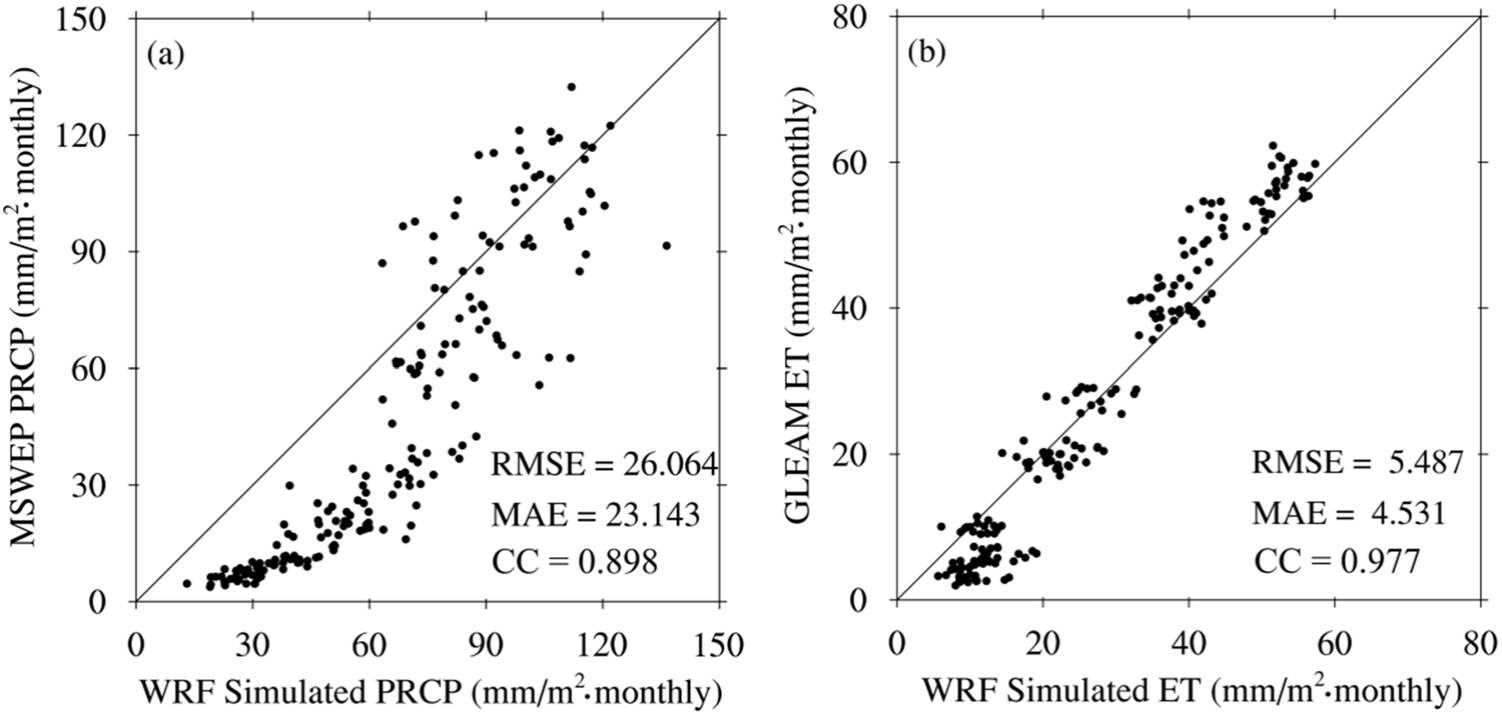
Comparison of precipitation (**a**) and ET (**b**) between the WRF simulation and observational data. The black dots represent the spatial averages over the TP for each month.

Figure 2 shows the comparison of precipitation and ET between the WRF simulation and observational data. The WRF-simulated precipitation is evaluated against the MSWEP precipitation observation product, yielding RMSE and MAE values of 26.064 mm/(m^2^ monthly) and 23.143 mm/(m^2^ monthly), respectively, with a correlation coefficient of 0.898. The precipitation simulated by WRF is generally higher than the MSWEP precipitation observation product. The RMSE and MAE between WRF-simulated ET and GLEAM ET product are 5.487 mm/(m^2^·monthly) and 4.531 mm/(m^2^·monthly), respectively, with a correlation coefficient of 0.977. ET simulated by the WRF model agrees well with GLEAM retrieved from satellite data. The validation results suggest that WRF demonstrates promising potential in simulating precipitation and ET, which are crucial components of the atmospheric water cycle on the TP. Therefore, the WRF model can be used for downscaling analysis on the TP, and the simulated data can be used to analyze the atmospheric water cycle processes and characteristics of the TP.

Although the WRF model has shown great simulation performance in reproducing the changes in precipitation and ET on the TP, there are still deviations between the simulation results of the WRF model and the observation data. As precipitation is the result of complex physical and dynamic processes, similar to most regional climate models, the WRF model tends to overestimate precipitation on the TP, leading to a significant wet bias. The choice to utilize the Kain–Fritsch scheme in the inner domain suggests considerations based on computational limitations. Parameterized convection offers a computationally efficient alternative for simulation. However, it is important to acknowledge that the use of convection parameterization introduces uncertainties. The resolution of the simulations, at 0.05°, lies within a gray zone where the applicability of convection parameterization schemes becomes less certain^51^. This can impact the accurate representation of small-scale convective processes and spatial heterogeneity. While the Kain–Fritsch scheme was chosen in this study, it is worth noting that other parameterization schemes or explicit convection simulations may yield different results.

Different reanalysis datasets can exhibit variations in representing atmospheric conditions, and the use of alternative datasets may yield different simulation results. The final operational global analysis data from the NCEP-FNL^52–54^ and ERA-interim from the European Centre for Medium-Range Weather Forecasts (ECMWF)^55–57^ are commonly used reanalysis datasets as the initial and boundary conditions in simulations. When using the NCEP-FNL data, the precipitation over the TP has been well represented^58^. Therefore, in this study, the NCEP-FNL reanalysis datasets were selected as the initial and boundary conditions. However, it is important to acknowledge that different reanalysis datasets can introduce uncertainty into the simulation results.

The estimation of precipitation over the TP remains challenging, and the selected precipitation analysis may be influenced by the spatial uncertainties, which refers to the variability and bias in simulating precipitation distribution in different geographical locations over the TP. These uncertainties arise from various factors, including simulation uncertainties, observational uncertainties, and the complex terrain of the Tibetan Plateau, which may influence water vapor transport and further increase the spatial uncertainty of precipitation distribution. Moreover, there are various ET products available, and their variability would influence the evaluation of model performance. In this study, the assessment of precipitation employed the MSWEP datasets, while the evaluation of ET utilized the GLEAM datasets. Liu et al.^59^ evaluated the accuracy of two daily precipitation products, CHIRPS (Climate Hazards Group Infrared Precipitation with Stations data) and MSWEP, over the TP from 1981 to 2015 and found that the MSWEP performs better than CHIRPS in terms of CC (MSWEP is 0.44, CHIRPS is 0.23) and RMSE (MSWEP is 4.21 mm, CHIRPS is 5.03 mm), with higher detection capabilities on the TP. Liu^60^ evaluated three remotely sensed ET products, including GLEAM, ET product produced by Zhang et al.^61^, and ET product produced by CSIRO (Commonwealth Scientific and Industrial Research Organisation), against water balance-based reference ET across the TP, and found that the GLEAM performed the best in terms of the multi-year average and inter-annual variability of monthly ET on the TP. It should be noted that even though well-performing precipitation and ET products were selected for model evaluation, there may still exist uncertainties associated with them.

### Annual water vapor transport

**Figure 3:**
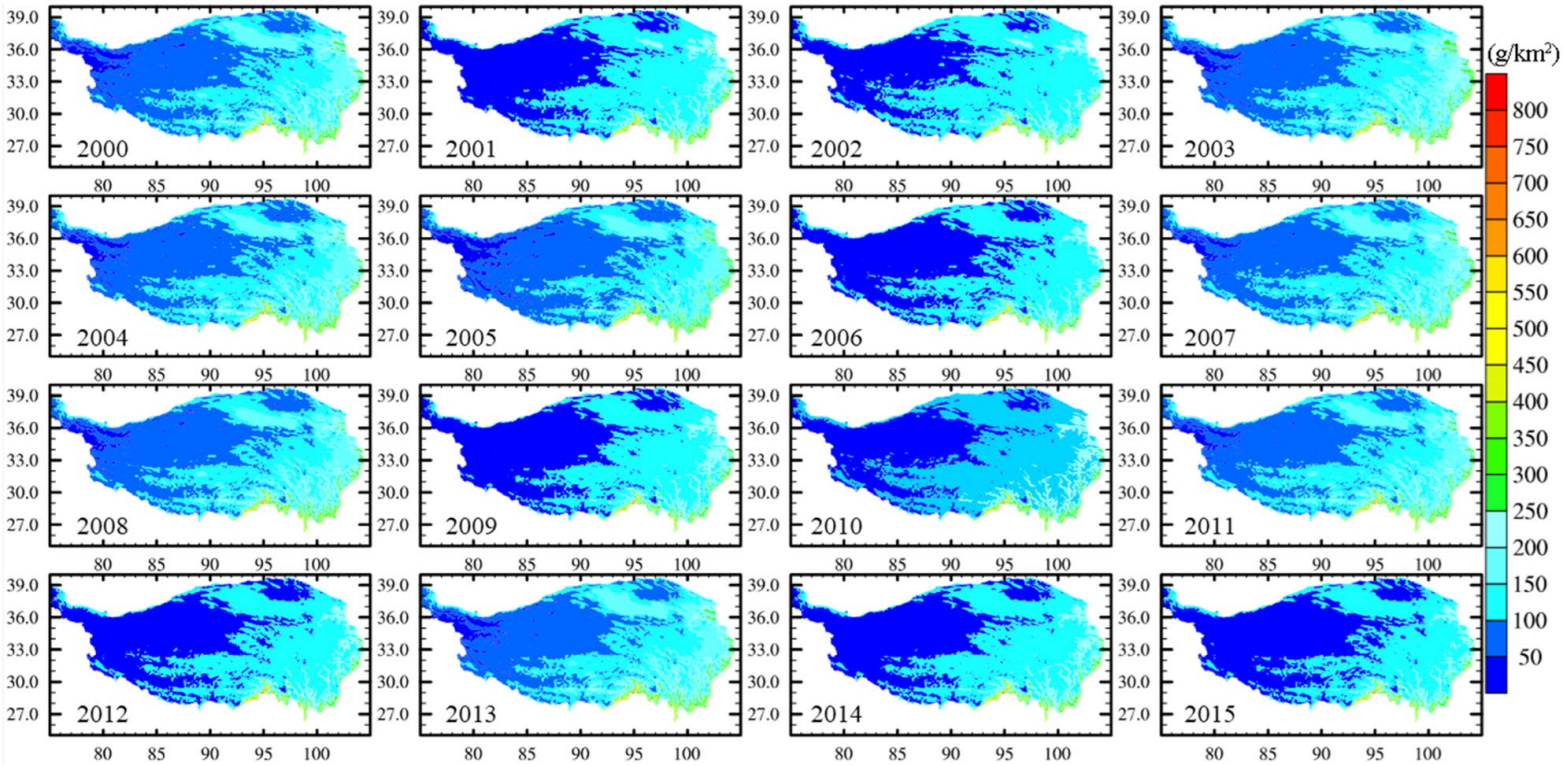
The annual mean total water path over the TP from 2000 to 2015 (g/km^2^).

Figure 3 shows the annual mean total water path of the TP from 2000 to 2015 calculated by the mixing ratios of the water vapor, cloud vapor, rainwater, ice water, and snow water. The total water path in the southern edge of the TP is the highest over the TP, and gradually decreases from southeast to northwest. The regions with the lowest total water path are located in the northwestern and northeastern parts of the TP. Furthermore, the interannual variability of annual mean total water path from 2000 to 2015 is not significant, especially in the southeastern region of the TP.

**Figure 4:**
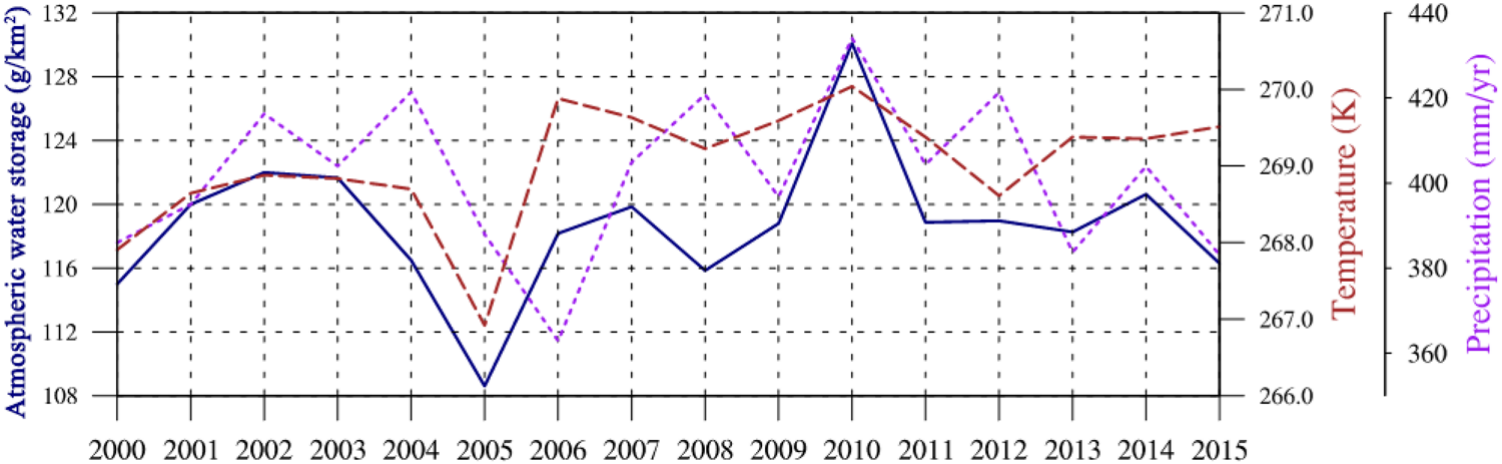
The time series of total water path, temperature, and precipitation from 2000 to 2015. The dark blue line, dark red line and purple line represent the total water path, temperature, and precipitation, respectively.

Figure 4 shows the time series of the regional mean total water path, temperature, and precipitation from 2000 to 2015. The total water path and temperature exhibit synchronous change trends. The changing trend of precipitation is consistent with that of the temperature and total water path, but it is delayed in time. For example, the lowest values of total water path and temperature appeared in 2005, followed by the lowest value of precipitation in 2006. Higher temperature increases the capacity to accommodate atmospheric water, and the precipitation will increase with the increase of atmospheric water. From 2000 to 2015, the temperature shows a fluctuating upward trend, whereas the increasing trends of total water path and precipitation are not significant.

**Figure 5:**
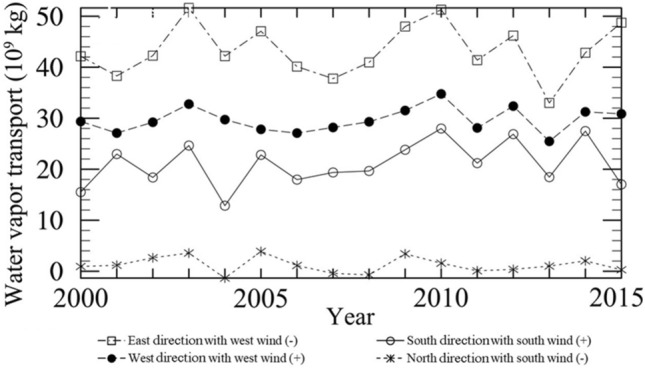
The annual total water vapor transport (10^9^ kg) from four directions based on WRF simulations. “East direction with west wind (-)” represents the annual total amount of water vapor flowing out from the eastern direction of the TP by west wind. “East direction with west wind (-)” represents the total amount of water vapor flowing in the TP by west wind from the western direction of the TP. “North direction with south wind (-)” represents the annual total amount of water vapor flowing out from the southern direction of the TP by south wind. “South direction with south wind (-)” represents the total amount of water vapor flowing in the TP by south wind from the southern direction of the TP.

Figure 5 shows the annual water vapor transport from four directions. The western and southern boundaries are the main sources of water vapor into the TP. The amount of water vapor transported into the TP from the western boundary, influenced by the mid-latitude westerlies, is greater than that from the southern boundary influenced by the south wind. However, a large amount of water vapor transported into the plateau from the west boundary flows out of the plateau from the eastern boundary of the TP, resulting in a negative net water vapor flux transported into the TP by mid-latitude westerlies. The water vapor in the TP mainly comes from the southern boundary, which is transported by the Indian summer monsoon from the southern boundary into the TP. The net water vapor flux transported from the southern boundary to the TP is positive. From 2000 to 2015, there is a slight increasing trend in the water vapor transported into the TP from its western and southern boundaries.

### Seasonal water vapor transport

#### Seasonal water vapor transport distribution

**Figure 6:**
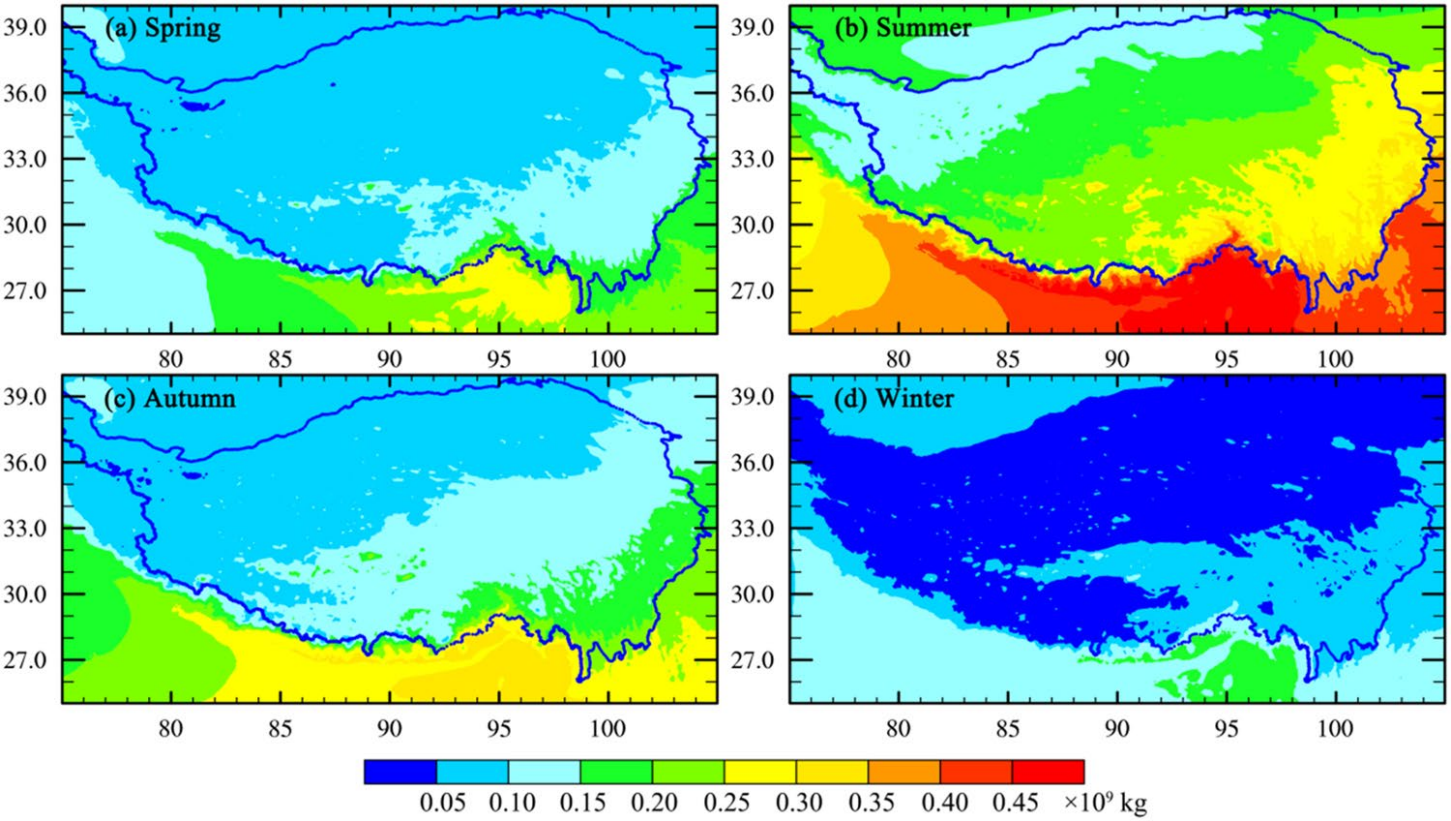
The mean seasonal water vapor distribution over the TP in Spring (**a**), Summer (**b**), Autumn (**c**), and Winter (**d**). The total column water vapor distribution is integrated by vertical integration from the surface to the top of the atmosphere for water vapor in different seasons.

Figure 6 shows that the water vapor distribution over the TP gradually decreases from southeast to northwest in different seasons. The highest water vapor content occurs in summer, while the lowest occurs in winter. In spring and autumn, the water vapor content is relatively similar. Specifically, in spring, the water vapor content ranges from 0.10 × 10^9^ to 0.15 × 10^9^ kg in the southeast region of the TP, while in most regions of the central and northwest parts of the TP, it ranges from 0.05 × 10^9^ to 0.10 × 10^9^ kg. In summer, the water vapor content is greater than 0.25 × 10^9^ kg in the southeast region of the TP, between 0.15–0.25 × 10^9^ kg in the central region of the TP, and less than 0.15 × 10^9^ kg in the northwest region of the TP. In autumn, the water vapor content ranges from 0.15 × 10^9^ to 0.25 × 10^9^ kg in the southeast of the TP, while in the northwestern of the TP, it is lowest, ranging from 0.05 × 10^9^ to 0.10 × 10^9^ kg. In winter, the water vapor content in most regions of the TP is less than 0.05 × 10^9^ kg. Overall, the water vapor content in the TP exhibits a significant decreasing trend from southeast to northwest, and the seasonal difference in water vapor content is significant. The spatial distribution of water vapor over the TP is attributed to several factors, including topography, and the location and intensity of fluxes in and out of the region. The TP is characterized by its high elevation, with the southeast being lower in altitude compared to the northwest. The topography plays a crucial role in influencing atmospheric circulation patterns, resulting in variations in moisture availability. As moist air masses encounter the TP, they are forced to ascend due to the higher terrain, leading to orographic lifting and enhanced condensation. This process contributes to the higher water vapor content in the southeast region compared to the northwest. Moisture advection from surrounding regions, like the Indian Ocean, reinforces the higher water vapor content in the southeast. Conversely, the moisture transport from the western mid-latitude westerlies results in most of the water vapor flowing out of the eastern boundary of the TP. These combined factors contribute to the spatial distribution of water vapor over the TP, with significant implications for regional precipitation patterns and the unique climatic characteristics in the TP.

#### Seasonal water vapor transport of four boundaries

**Figure 7:**
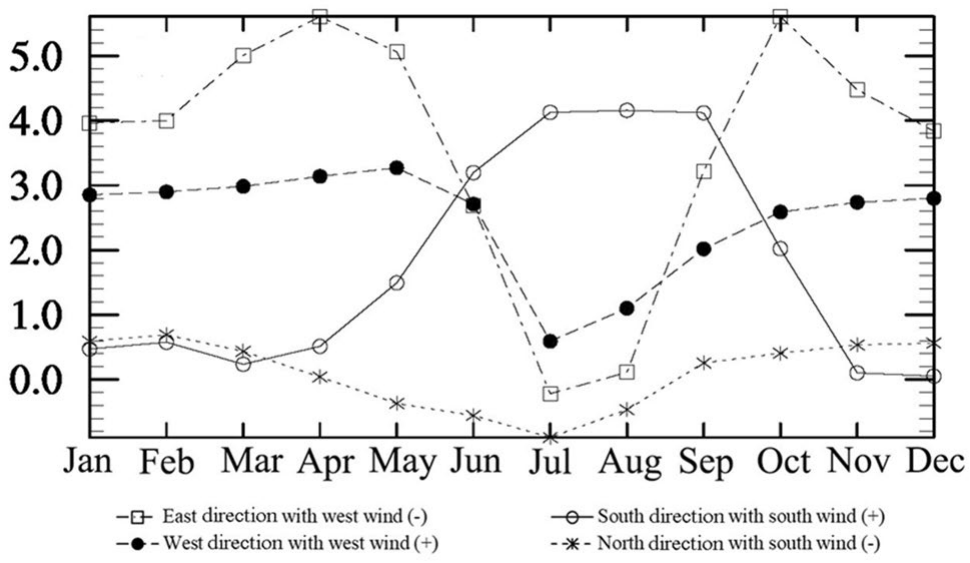
The seasonal water vapor transport (10^9^ kg) from four directions based on WRF simulations. “East direction with west wind (-)” represents the annual total amount of water vapor flowing out from the eastern direction of the TP by west wind. “East direction with west wind (-)” represents the total amount of water vapor flowing in the TP by west wind from the western direction of the TP. “North direction with south wind (-)” represents the annual total amount of water vapor flowing out from the southern direction of the TP by south wind. “South direction with south wind (-)” represents the total amount of water vapor flowing in the TP by south wind from the southern direction of the TP.

Figure 7 illustrates the seasonal water vapor transport from four directions of the TP. The water vapor is predominantly transported into the TP from its western and southern boundaries, which is in agreement with previous research results, that is, the water vapor of the TP is mainly transported by the Indian summer monsoon and mid-latitude westerlies^3–5^. The water vapor transported into the TP mainly flows out from the eastern boundary of the plateau, with very low outflow from its northern boundary. In spring, the water vapor transported into the TP is primarily influenced by the mid-latitude westerlies through its western boundary, while the water vapor also flows out of the TP from its eastern boundary under the influence of the west wind. Therefore, the net water vapor flux impacted by the mid-latitude westerlies is negative. The water vapor content flowing into the plateau from its southern boundary is low. In summer, the influence of the mid-latitude westerlies weakens, and the water vapor content transported by the west wind decreases significantly. The impact of the Indian summer monsoon on the water vapor transport process over the TP gradually strengthens and plays a key role. The water vapor over the TP mainly comes from the southern boundary transported by the Indian summer monsoon. In autumn, the water vapor transported by the Indian summer monsoon gradually decreases, while the water vapor transported by the mid-latitude westerlies increases. Under the influence of the west wind, most of the water vapor flows out of the TP from its eastern boundary. In winter, the water vapor content transported from the southern boundary into the TP further decreases. The water vapor of the TP is mainly transported from the western boundary to the TP due to the impact of the mid-latitude westerlies, and then part of the water vapor flows out of the TP from its eastern boundary under the influence of the west wind. The net water vapor in spring and winter is negative with a small amount, is far positive in summer, and is positive in autumn with a small amount either.

### Moisture recycling over the TP

**Figure 8:**
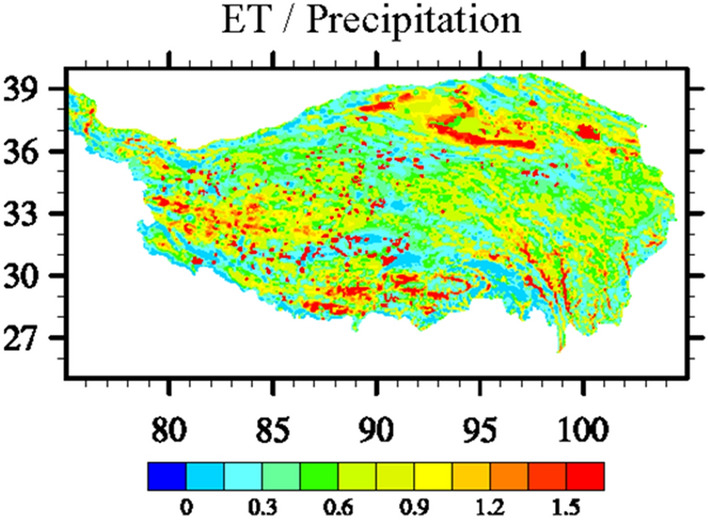
Spatial distribution of ratio of ET versus precipitation over the TP.

Table 2 shows the observed and simulated precipitation, ET, and the ratio of ET to precipitation. Based on the MSWEP and GLEAM, the monthly mean precipitation and ET flux over the TP are 45.10 mm/m^2^ and 27.83 mm/m^2^, respectively. Consequently, the ratio of ET to precipitation is 0.62. The WRF-simulated precipitation and ET fluxes over the TP are 64.53 mm/m^2^ and 31.20 mm/m^2^, respectively. Figure 8 shows the spatial distribution of the ratio of ET to precipitation over the TP. The ratio of ET versus precipitation over the TP is approximately 0.48. It is important to note that this ratio can not fully represent the regional moisture recycling, as a portion of the water vapor from ET may flow out the study area and contribute to precipitation elsewhere, and the water vapor that forms precipitation also includes water vapor transported to the Tibetan Plateau from external sources. According to Eq. (3), i.e., $\gamma =\frac{P-{C}_{q}}{P}$, the regional moisture recycling rate over the TP is estimated to be approximately 0.37.

**Table 2:** Observed and simulated precipitation, ET, and the ratio of ET versus precipitation.

Item	Monthly flux (mm/m^2^)	Ratio (ET/P)
TP-Precipitation (MSWEP)	45.10	0.62
TP-ET (GLEAM)	27.83	
TP-Precipitation (WRF)	64.53	0.48
TP-ET (WRF)	31.20

## Discussion

### Water vapor transport over the TP

Previous studies have confirmed that the water vapor on the TP is mainly transported into the TP from its southern and western boundaries, with water vapor from the Indian Ocean and the southern edge of the Bay of Bengal dominating the summer precipitation of the southeastern TP, and the southern boundary of the TP serves as the main water vapor pathway^23,62^. In addition, Curio et al.^22^ proposed that the mid-latitude westerlies are not completely blocked by the TP, and their contribution to summer water vapor transport is higher than previously assumed, but the mountainous terrain still significantly restricts the process of water vapor transport. There is still controversy over the water vapor transport process on the TP and the contribution of water vapor from different sources.

Numerous studies have shown that the western and southern boundaries of the TP are the main inflow boundaries of water vapor, while the eastern boundary of the TP is the main outflow boundary of water vapor. Based on ERA5 reanalysis data, Yu et al.^63^ demonstrated that the amount of water vapor entering the TP from its southern boundary significantly surpassed that entering from the western boundary. However, in this study, high-resolution simulations were conducted to compute water vapor fluxes through four boundaries. Our results indicated that the water vapor entering the TP from the western boundary surpassed that from the southern boundary. The water vapor transported from the southern boundary of the TP might be lower than previously anticipated. These results may potentially be attributed to the refined description of the complex topography by high-resolution simulations, which might affect the actual water vapor transport from its southern boundary. Furthermore, both the simulations in this study and reanalysis data indicated a negative net water vapor flux in the east–west direction and a positive net flux in the north–south direction across the TP.

The water vapor transport processes on the TP show significant differences in different seasons. In spring, the mid-latitude westerlies have a greater impact on the water vapor transport process, with water vapor mainly coming from the western boundary of the plateau and flowing out of the eastern boundary, resulting in a negative net water vapor flux. In summer, the influence of the mid-latitude westerlies on the water vapor transport process weakens, but the amount of water vapor transported into the TP from the western boundary is greater than that flowing out of the eastern boundary, resulting in a positive net water vapor flux. A large amount of water vapor is transported into the TP from the southern boundary under the influence of the Indian summer monsoon, making the TP a water vapor sink in summer. In autumn, the influence of the Indian summer monsoon on the water vapor transport process weakens, while the influence of the mid-latitude westerlies strengthens, with water vapor mainly entering the TP from the western boundary and exiting from the eastern boundary. In winter, the water vapor transport process on the TP is mainly influenced by the mid-latitude westerlies.

In addition, from 2000 to 2015, there is an increasing trend in total water path, with the lowest value in 2005 at approximately 109 g/km^2^ and the highest value in 2010 at around 130 g/km^2^ (Fig. 4). These findings are consistent with previous research. He et al.^15^ analyzed the atmospheric water cycle processes over the TP using ERA5 reanalysis data from 1979 to 2019, and found an increasing trend in total water path over the TP, with the external transport contributed to 36.13% of the increase in water vapor. Li et al.^64^ used the ERA-Interim, MERRA-2, and JRA-55 reanalysis data to analyze the atmospheric water vapor transport processes over the endorheic TP from 1979 to 2015, and found that atmospheric water originating from the land dominates the annual-scale contribution to total water path. The increasing trend in total water path indicates that the atmospheric water cycle process is intensifying in the context of climate warming.

This study clarifies the influence of the mid-latitude westerlies and the Indian summer monsoon on the water vapor transport processes over the TP and assesses its inter-annual and seasonal characteristics. It is worth noting that there are several high-resolution regional climate simulation datasets available for the TP^54,65–68^. However, compared to existing datasets like Maussion et al.^54^ which provide high-resolution climate simulations for the TP at resolution of 10 km, the datasets used in this study covers the period from 2000 to 2015 at resolution of 0.05°. The higher resolution datasets allow for a more detailed representation of the complex terrain over the TP^35,69^, reducing biases in precipitation and better capturing the atmospheric water cycle processes over the TP. This study provides a comprehensive assessment of the interplay between the mid-latitude westerlies and the Indian summer monsoon in influencing the water vapor fluxes over the TP. However, the primary water vapor sources on the TP and their contributions have not been investigated. In the future, it is necessary to combine different methods and data to further analyze the water vapor sources of the TP and their contributions.

### Water vapor sources to TP in summer

The TP, often referred to as the AWT, is not only significant due to its abundant frozen water reserves, but also for its role as a critical supplier of atmospheric water vapor. A large amount of water vapor is transported from the TP to the surrounding areas via the eastern edge. Therefore, the TP is aptly termed the AWT for its vital role in supplying and regulating both surface and atmospheric water.

However, which one of four-wards plays as the dominant water vapor source over TP remains divergent, especially in summer. Yan et al. (2020) pointed out that water flux trends in eastern and northern boundaries are much higher than those in western and southern boundaries during summer based on ERA5 (1979–2018), which is totally different from the result in this study shown in Fig. 7. Meanwhile, Lin et al.^70^ investigated water vapor transport in summer from 1979 to 2014, and proposed that the water vapor transport of the AWT mainly came from its southern, southwestern, western, and northern boundaries, with the southern boundary playing the most crucial role. Chen et al.^71^ also pointed out that the southern, northern, and western boundaries of the AWT serve as the primary inputs for water vapor, with the southern boundary in summer contributing 38.8% of the annual amount, while the eastern boundary acts as an output boundary. Similarly, Xie et al.^72^ concluded that the main source of water vapor for the AWT is the southern boundary, with an outward flow along the eastern boundary. Furthermore, using the ERA-interim and JRA55 monthly reanalysis datasets, Xu et al.^73^ investigated the climatic characteristics of water vapor transport over the TP from 1979 to 2018 and found that the primary sources of water vapor are the southern and western boundaries of the TP, water vapor transported from the southern boundary is traced back to the Arabian Sea and the Bay of Bengal, while that from the western boundary is attributed to mid-latitude westerlies.

The findings of this study are consistent with the most previous studies, showing that the water vapor primarily enters the AWT through its southern and western boundaries in summer, with the largest contribution transported from the southern boundary in summer. While variations in results may arise due to different methodologies and datasets, it is generally acknowledged that the southern and western boundaries of the TP contribute significantly to water vapor supply in summer.

### Physical mechanism of water vapor transport

The physical mechanism of water vapor transport over the TP is complex^23^. The large-scale atmospheric circulations play dominant roles in the process of water vapor transport, including the mid-latitude westerlies and the Indian monsoon system, thereby regulating the interannual variations of precipitation over the TP^73^. In addition, the high topography of the TP is considered both a barrier to the mid-latitude westerlies and an enhancer of the Indian summer monsoon through its dynamical and thermal driving forces, thereby promoting the large-scale atmospheric circulation^5^. Therefore, topography plays a crucial role in affecting the water vapor transport processes over the TP, and a refined representation of topography helps improve the representation of the physical processes and reduce biases in precipitation simulations^35^. It’s worth noting that climate teleconnections, such as the Indian Ocean Dipole and the El Niño-Southern Oscillation, also influence the water vapor transport processes^17,74^. These phenomena affect sea surface temperatures and, consequently, impact the behavior of the Indian summer monsoon, and changes in the monsoon’s intensity and timing can alter the amount and distribution of water vapor transported to the TP^62,70,75^.

It is important to understand the physical mechanisms behind water vapor transport over the TP for accurately modeling regional climate and predicting precipitation. Future research should delve deeper into these mechanisms to enhance the comprehension of the atmospheric water cycle over the TP and its broader implications for the Asian continent, which is invaluable for water resource management, agriculture, and disaster preparedness in this region.

### Limitation

The novel method to calculate the water vapor transport along the elevation boundary, should be done the same way for other input data (e.g. ERA5) to see the higher-resolution topography impacts the water vapor transport into the Tibetan Plateau. However, due to page limitation, we are unable to include this comparison in the current manuscript and will conduct such comparison analysis in future work.

## Conclusion

In this study, using the WRF model, we conducted a detailed analysis of water vapor transport processes over the TP through high-resolution simulations, providing the basis for the assessment of the impact of climate change. High-resolution modeling allows for a detailed analysis of water cycle processes and further assessment of simulation reliability and uncertainty, particularly in reproducing water vapor transport patterns. Understanding the WRF model’s capability to capture current climate conditions contributes to assessing the reliability of predicting changes in water vapor transport processes due to climate warming in the future. Additionally, this study provides insights into the characteristics of water vapor transport at different boundaries of the TP, offering important insights for future exploration of the impact of global warming on water vapor transport mechanisms. The main conclusions are as follows:

1. The total water path of the TP gradually decreases from the southeast to the northwest, with the highest total water path located at the southern edge of the TP, and the lowest in the northwest and northeast regions. From 2000 to 2015, the temperature of the TP shows a fluctuating upward trend, and the total water path and temperature showed synchronous changes, with higher temperatures increasing the atmospheric water vapor holding capacity. The changes in precipitation are consistent with the changing trend of total water path but lag behind the changes in total water path over time.
2. The water vapor over the TP is primarily transported from the western and southern boundaries and flows out through the eastern boundary, with very little flowing out through the northern boundary, which is consistent with the previous findings of water vapor transport in this region, as the high topography of the TP hinder the transportation of water vapor from its northern boundaries. The amount of water vapor transported into the TP from the western boundary by west wind is higher than that transported from the southern boundary by south wind. However, most of the water vapor flows out of the TP through the eastern boundary under the influence of the west wind, resulting in a negative net water vapor flux transported by west wind. Water vapor transported from the southern boundary contributes more to precipitation on the TP. It is worth noting that the relationship between water vapor transport and different atmospheric circulation systems should be further investigated.
3. For seasonal differences, the water vapor content is highest in summer and lowest in winter, while the water vapor content is similar in spring and autumn. In spring, water vapor is mainly transported into the TP from the western boundary by mid-latitude westerlies, but most of the water vapor flows out through the eastern boundary, resulting in a negative net water vapor flux influenced by the westerlies. In summer, the water vapor transported by mid-latitude westerlies weakens, while the water vapor transported by the Indian summer monsoon strengthens. Water vapor is mainly transported into the TP from the southern boundary, contributing a large amount of water vapor to summer precipitation on the TP. In autumn, the water vapor transport process controlled by mid-latitude westerlies gradually strengthens, while that controlled by the Indian summer monsoon gradually weakens. Water vapor is mainly transported into the TP from the western boundary and flows out through the eastern boundary. The change process in winter is similar to that in spring.
4. The moisture recycling rate is about 0.37, and the ratio of ET to precipitation is approximately 0.48. Therefore, external water vapor transport is the primary source of water vapor on the TP, but the contribution of locally recycled moisture cannot be ignored. Especially in the context of global climate change, significant changes in the surface environment of the TP have enhanced regional ET, increasing the uncertainty of the atmospheric water cycle process on the TP.

This study focuses on the changes in the inflow and outflow of water vapor from the four boundaries of the TP. Due to the complex terrain of the TP, it is necessary to conduct higher resolution simulations in future research to further analyze the characteristics of water vapor transport and the changes in moisture recycling on the TP.

## Data Availability

The processed simulation datasets and materials used in this study are available upon request to the first author (panxd@itpcas.ac.cn). The input data for the WRF model were obtained from the Research Data Archive (RDA), which is maintained by the Computational and Information Systems Laboratory (CISL) at the National Center for Atmospheric Research (NCAR). The original data are available from the RDA (http://rda.ucar.edu/datasets/ds083.2/, accessed on 10 July 2019) in data set number ds083.2. The validation datasets are downloaded from open sources. MSWEP datasets are available from https://www.gloh2o.org/. GLEAM datasets are available from https://www.gleam.eu/.
